# Crosstalk between Heart Failure and Cognitive Impairment via hsa-miR-933/RELB/CCL21 Pathway

**DOI:** 10.1155/2021/2291899

**Published:** 2021-09-18

**Authors:** Wenxiao Feng, Jie Yang, Wenchao Song, Yitao Xue

**Affiliations:** ^1^First Clinical Medical College, Shandong University of Traditional Chinese Medicine, Shandong, China; ^2^Jinan Changqing District People's Hospital, China; ^3^Department of Cardiovascular, Affiliated Hospital of Shandong University of Traditional Chinese Medicine, Shandong, China; ^4^Department of Encephalopathy, Zouping Hospital of Traditional Chinese Medicine, Shandong, China

## Abstract

**Background:**

The association between heart failure (HF) and cognitive impairment has received increasing attention from scholars and researchers in recent years. However, no systematic studies have been carried out yet focused on the crosstalk between heart failure and cognitive impairment via miRNAs.

**Methods:**

GSE104150, GSE53473, GSE120584, and GSE116250 with RNA-seq data and clinical data were downloaded from the GSE database. All data were statistically analysed using R software to detect DE-miRNAs and DE-mRNAs associated with both HF and cognitive impairment. Protein-protein interaction (PPI) networks were mapped, and a logistic regression model for cognitive impairment prediction was developed. Furthermore, the TTRUST database and miRWalk were used to map miRNA-transcription factor (TF) and messenger RNA (mRNA) regulatory pathways. Finally, core TFs were enriched for analysis.

**Results:**

Differentially enriched DE-miRNAs and DE-mRNAs both present in HF and cognitive impairment were determined. A logistic regression model established based on DE-miRNAs was validated to have a strong performance in cognitive impairment prediction. The core miRNA-TF-mRNA pathway was formed by mapping the PPI networks associated with the two diseases. Further GSEA was performed with V-rel reticuloendotheliosis viral oncogene homolog B (RELB) as the core TF, and the retinol metabolism and gap junction pathways were analysed.

**Conclusions:**

This study was the first attempt to predict the crosstalk and examine underlying mechanisms between HF and cognitive impairment applying bioinformatics. The findings suggested a potential hsa-miR-933/RELB/CCL21 regulatory axis correlated with HF and neurological disorders (or cognitive impairment), according to PPI networks.

## 1. Introduction

Chronic heart failure (HF), alternatively known as chronic congestive heart failure, is the most advanced form of most cardiovascular diseases and a primary cause leading to patients' death. HF has largely resulted from myocardial damage caused by myocardial infarction, cardiomyopathy, haemodynamic overload, or inflammation. HF will weaken the contractility of heart muscles, preventing the maintenance of normal cardiac output [1]. The prevalence of HF in the general population is about 1-2%, but it exceeds 10% among the elderly aged over 70 [2, 3]. Statistics estimated that the number of patients suffering from cognitive impairment will reach around 74.7 million worldwide by 2030 [4]. Interestingly, approximately 25-75% of HF patients are accompanied by cognitive impairment [5]. Such a potential association between HF and cognitive impairment has attracted growing research attention [6].

Currently, though clinical indicators related to chronic HF have been identified, we still face a lack of more sensitive and accurate markers for early diagnosis, treatment, and prognostic assessment of chronic HF. miRNAs are small, endogenous RNAs of approximately 20 to 24 nucleotides in length with important regulatory roles in cells. miRNAs are involved in various pathological and physiological processes during HF development and cognitive impairment [7–10]. Disease diagnosis and prediction of prognosis could be based on testing certain miRNAs, as different compositions of miRNA components are often indicative of disease types [11]. For example, as one of the most widely studied miRNAs, miR-206 has been identified to be closely associated with the development of HF and cognitive impairment [12]. HF is also related to changes in the microenvironment of the circulatory system [13]. Interestingly, these changes are present in cognitive impairment progression [14]. However, so far, no previous research has investigated the potential crosstalk between HF and cognitive impairment via miRNAs.

Bioinformatics allows the detection of specific signaling pathways via which diseases occur and analysis on the disease-target correlation, showing a strong potential of its use in predicting target mRNA binding sites potentially associated with cognitive impairment and HF. The present research is aimed at investigating the relationship between HF and cognitive impairment based on miRNA crosstalk and the underlying mechanisms, hoping to provide a theoretical basis for clinical translation.

## 2. Materials and Methods

### 2.1. Data Collection and Preprocessing

Microarray data was downloaded from GEO (http://www.ncbi.nlm.nih.gov/geo/). Microarray data of miRNA expression in peripheral blood derived from HF patients were downloaded from GSE104150 [15] and GSE53473 [16], while those of cognitive impairment patients were downloaded from GSE120584 [17]. Subsequently, the RNA-seq data of HF myocardial tissues were acquired from GSE116250 [18] to obtain mRNA expression. mRNA expression microarray data in brain tissues of cognitive impairment came from GSE140831 [19]. The GSE120584 dataset contained serum miRNA and corresponding clinical data of 1569 cases of cognitive impairment patients and normal controls; the GSE116250 dataset contained the mRNA expression data of 64 HF patients and normal controls; the GSE140831 dataset contained the mRNA expression data of 1129 patients with cognitive impairment and normal controls. Corresponding clinical information of patients in each GEO dataset was downloaded. RT-PCR data of the corresponding miRNAs in the predictive model were acquired. In addition, the corresponding clinical data were obtained. Informed consent was gained. Finally, RT-PCR data and clinical data from 95 patients who attended our hospital were acquired and served as a test set to evaluate the performance of the prediction model. The study procedure was reviewed and approved by the local ethical committee.

### 2.2. Analysis of Variances

The miRNA and mRNA microarray gene IDs were converted to gene symbols based on the microarray platform files. According to data types and sizes, the expression matrix was further log-transformed to obtain the miRNA and mRNA gene expression matrix. The limma [20] package was used to remove batch effects from the merged datasets when the same type of data was obtained from different platforms.

The Fragments Per Kilobase Million (FPKM) and Transcripts Per Kilobase Million (TPM) type data were analysed to reveal mRNA/miRNA differences between HF and healthy control (HC) and between cognitive impairment and HC according to the clinical sample grouping using the limma package. Differentially expressed RNA between the two groups was filtered based on a threshold value. ∣FC > 1.1∣ was the threshold of miRNA differential analysis. DE-miRNAs and DE-mRNAs of HF and HC, as well as cognitive impairment and HC, were intersected to obtain common DE-miRNAs and DE-mRNAs. The results were presented in a Venn diagram using the VennDiagram package.

### 2.3. miRNA-TF-mRNA and PPI Network and Subnetwork Construction

TF and its target mRNA were acquired from the TTRUST database. The TF and corresponding mRNAs in the shared DE-mRNA database were compared to develop a DE-TF-mRNA regulatory relationship network. Target mRNAs of the shared DE-miRNAs were predicted by the miRWalk (http://mirwalk.umm.uni-heidelberg.de/) database. The results were analysed with the DE-TF-mRNAs to acquire the intersection of the miRNA-TF/mRNA relationship network. Cytoscape (version 3.8.2) was employed for the visualisation of the miRNA-TF-mRNA regulatory PPI network. Finally, the MCODE plugin (degree cutoff = 2, node score cutoff = 0.2, *k*‐core = 2, and max.depth = 100) was used to create an aggregation of core genes and subnetworks in the network. The top two core subnetworks were selected according to the enrichment score, and their relational pairs and nodes were acquired for further analysis.

### 2.4. Construction of a Logistic Regression Model to Predict the Incidence of Cognitive Impairment Patterns

Shared DE-miRNAs and clinical information were analysed by univariate and multivariate logistic regression analyses. Factors from the common dataset and public database predictive of cognitive impairment onset were screened to develop two prediction models by logistic regression. A nomogram was created using the “rms” package to calculate and predict cognitive impairment incidence. Furthermore, a calibration curve was plotted to determine the calibration of the model. Public dataset and clinically acquired data served as the training group and the test group, respectively, for model training. The receiver operating characteristic (ROC) curve was plotted using the ROCR package [21] and the Hmisc package, and the *C*-index was calculated to assess the predictive and discriminatory performance of the model in the training, overall, and test groups. A *C*-index between 0.7 and 1 represented a high predictive performance. Finally, to evaluate the prediction range of the model, decision curves were plotted using the “rmda” package. The package was also used to assess the nomogram as well as clinical applicability and safety of the model.

### 2.5. Thermal and Volcanic Mapping

Heatmap and volcano maps of the DE-miRNAs and their target mRNAs in the prediction model for cognitive impairment and for normal controls were, respectively, intersected using the pheatmap and gplots packages. Furthermore, the miRNAs and target mRNAs shared by both the core genes of the PPI subnetwork and the prediction model were marked in the volcano map. The ggalluvial and ggplot2 packages were employed to develop a mulberry map of differential miRNA-TF/mRNA axes, which were considered to play a regulatory role in the pathogenesis of cognitive impairment.

### 2.6. Functional Enrichment Analysis and Statistical Analysis

Functional enrichment analysis was carried out using FunRich (v.3.1.3) for DE-miRNAs shared by both HF and cognitive impairment. Upregulated and downregulated genes were identified from the differential mRNAs targeted by key miRNAs in the prediction model for cognitive impairment and further subjected to functional GO and KEGG pathway enrichment mapping. Enrichment analysis of GO and KEGG pathways was conducted using the org.Hs.eg.db and clusterProfiler packages, and bar graphs were created [22]. The pathways were filtered by *P* value (<0.05). The TF factors were selected according to the fold change value and enriched by GSEA software (version 4.1.0), based on the obtained miRNA-TF-mRNA axis. The gene set “c2.cp.kegg.v7.4.symbols.gmt” was used for pathway enrichment annotation.

Statistical analysis was performed in R software (version 4.0.5). Hypothesis testing was conducted using a two-sided test.

## 3. Results

### 3.1. Analysis of Variances and Network Construction

**Figure 1:**
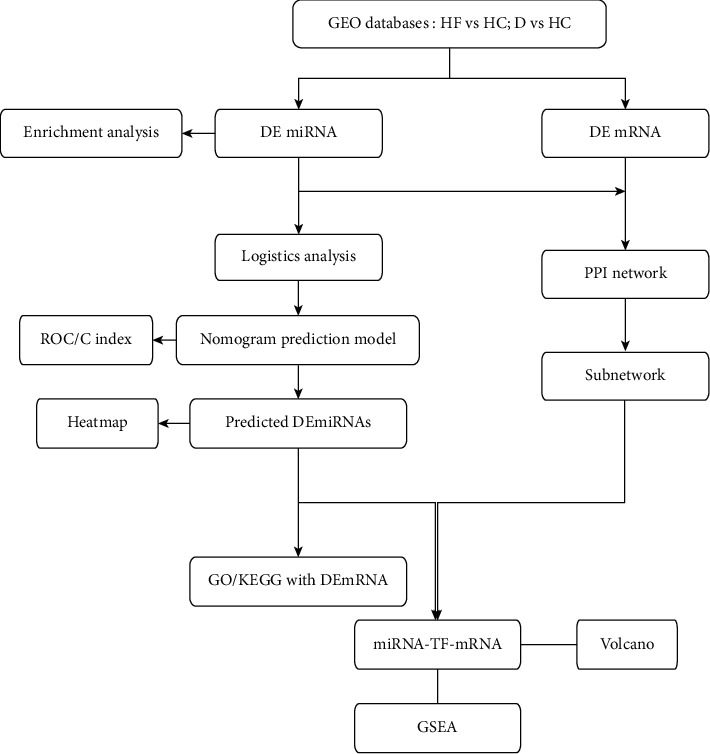
Flow chart of the analysis process.

The flow chart of the study analysis is shown in Figure 1. DE-miRNA and DE-mRNA were acquired from the downloaded datasets, and miRNA-TF/mRNA network and cognitive impairment prediction model were built. Finally, the core miRNAs and TFs were analysed.

**Figure 2:**
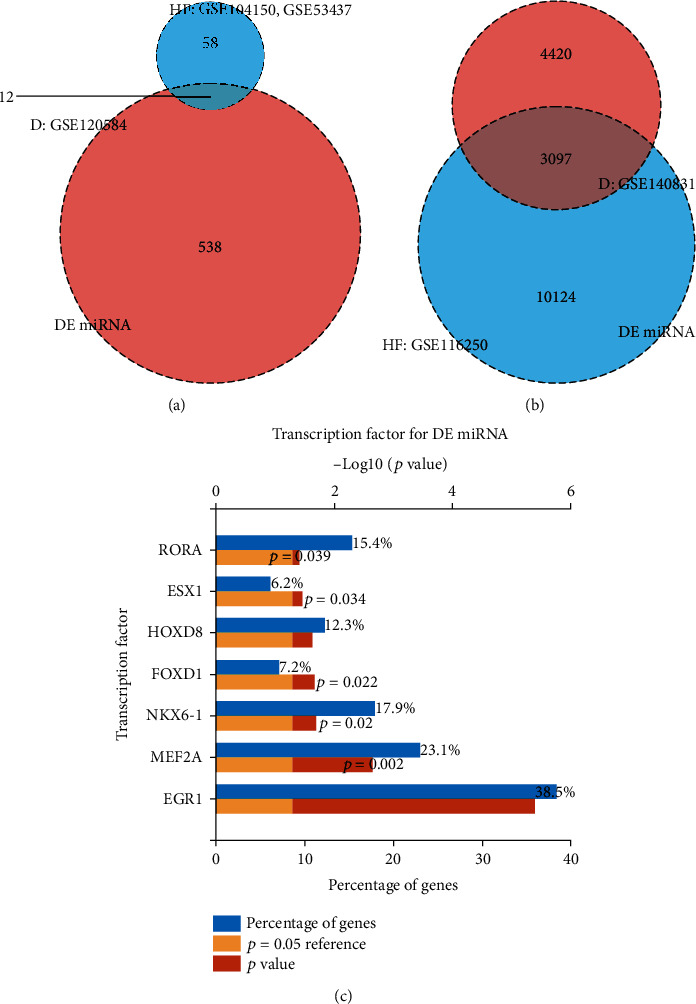
Venn diagram of mRNA and miRNA differential analysis results and differential miRNA pathway enrichment analysis. (a) Venn diagram shows the number of DE-miRNAs in HF and cognitive impairment, respectively, and the common differentially expressed miRNAs. (b) Venn diagram depicts the number of DE-mRNAs in HF and cognitive impairment, respectively, and the common differentially expressed mRNAs. (c) The bar chart exhibits the 11 DE-miRNAs targeted to TFs in cognitive impairment.

The Venn diagrams showed DE-miRNAs (including hsa-miR-342-3p, hsa-miR-1246, hsa-miR-615-3p, hsa-miR-1224-5p, hsa-miR-636, hsa-miR-1257, hsa-miR-551a, hsa-miR-486-5p, hsa-miR-485-3p, hsa-miR-933, and hsa-miR-296-3p) incorporating 3097 common DE-mRNAs in HF and cognitive impairment (see Figures 2(a) and 2(b)). Functional enrichment analysis of DE-miRNA transcription factors showed enrichment in EGR1, MEF2A, NKX6-1, FOXD1, ESX1, and RORA (see Figure 2(c)).  Table 1 exhibits the enrichment of DE-miRNAs in biological processes, cellular components, molecular functions, and biological pathways.

**Figure 3:**
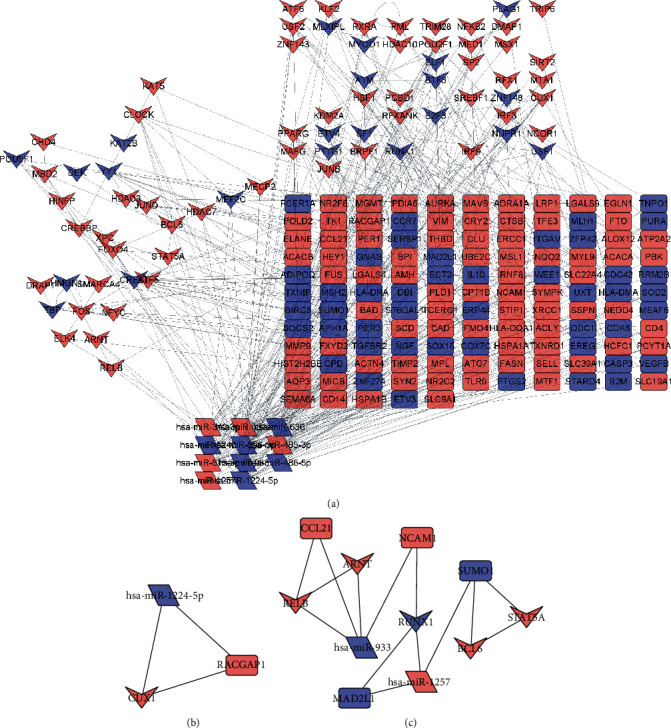
Protein interaction networks mapped from DE-miRNA, DE-mRNA, and predicted miRNA target gene prediction sites. (a) miRNA-TF-mRNA-regulated protein interaction network. (b, c) The top 2 aggregator network modules predicted according to MCODE. It represents the core group of genes involved in the disease process. The rectangles represent general protein mRNAs, the inner quadrilateral represents TF, and the rhombus represents miRNAs.

Figure 3(a) displays the PPI mapping and construction of regulatory networks for DE-miRNAs and their predicted target DE-mRNAs. Based on the MCODE and filtering conditions, the top two aggregated subnetworks and core genes were obtained (see Figures 3(b) and 3(c)).

### 3.2. Single and Multifactor Logistic Regression Analyses

In cognitive impairment and normal control datasets, univariate and multivariate logistic regression analyses were performed on the above 11 DE-miRNAs together with two important clinical factors age and gender. The results of the univariate analysis were filtered by *P* value (<0.05) and showed statistical significance of the factors (see Table 2). Moreover, all the factors were subjected to multifactorial analysis. Here, we found that age, sex, hsa-miR-485-3p, hsa-miR-486-5p, hsa-miR-933, hsa-miR-551a, and hsa-miR-1224-5p were independent predictors.

### 3.3. Logistic Regression Model Construction and Testing

**Figure 4:**
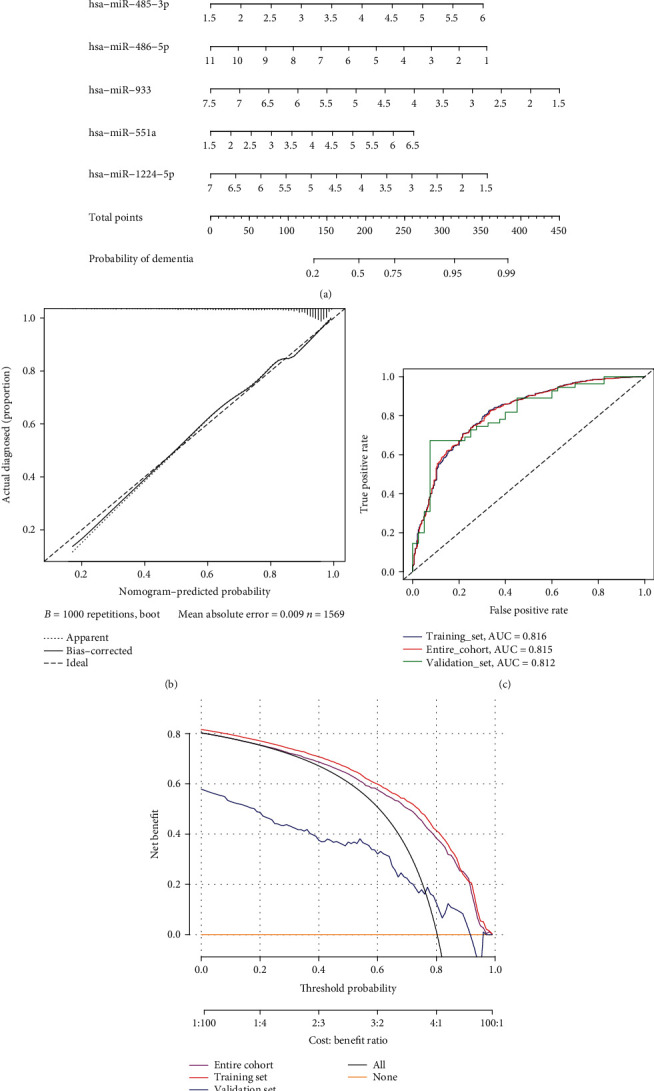
Logistic model based on DE-miRNA prediction of cognitive impairment. (a) Nomogram shows column plots for calculating the predicted incidence of cognitive impairment. (b) The calibration plot shows the calibration of the prediction results of the model. (c) The ROC curves show the classification and prediction performance of the prediction models. The training group, the clinical test group, and the overall group were evaluated separately. (d) Reliable range of model prediction probabilities demonstrated by DCA curves.

Table 3 shows the weights of the coefficients in the constructed model and the results of the statistical test of variance. Based on the constructed logistic regression model, the nomogram was plotted as a predictive model (see Figure 4(a)). Information on clinical patient characteristics was shown in Supplementary Table 1, and these data were the validation group for the predictive model.  Figure 4(b) shows the calibration assessment of the predictive model in a calibration graph.  Figure 4(c) shows the ROC and AUC of the model in the training, test, and overall groups.  Table 4 displays the *C*-index values that ranged from 0.812 to 0.816 for the three groups. This indicated that the model had good predictive classification performance.  Figure 4(d) shows a wide clinical applicability and high safety of the prediction model in the training, test, and overall groups, according to the decision curve analysis (DCA) curves.

### 3.4. Heatmap of DE-miRNA and Targeted DE-mRNA in Logistic Prediction Model

**Figure 5:**
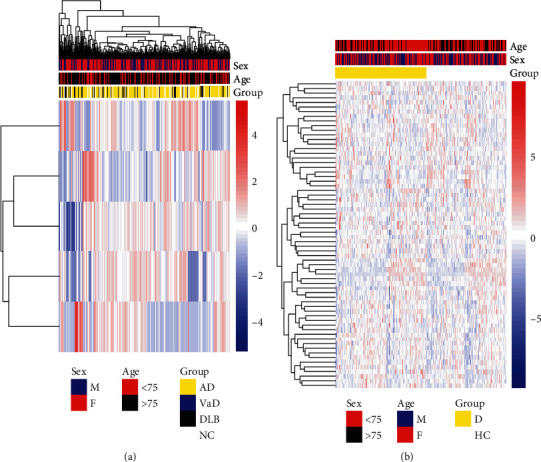
Heatmap of DE-miRNAs' factor in predicting cognitive impairment and the expression of their targeted DE-mRNAs in the models. (a) Heatmap of DE-miRNA expression shows the expression of 5 DE-miRNAs predictive of cognitive impairment in the prediction model. (b) Heatmap of DE-mRNA expression shows the expression of DE-mRNA targeted by the five predictors in the model of patients with cognitive impairment, Alzheimer's disease (AD), vascular dementia (VaD), Dementia with Lewy Bodies (DLB), Mild Cognitive Impairment (MCI), and dementia (D).

Based on the coefficients of the prediction model, the expression of five of the miRNAs in each cognitive impairment group and normal samples was presented as a heatmap (see Figure 5(a)). In relation to overall miRNA expression, factors such as age and gender were also shown. Based on the previously calculated PPI network, the expression of the target DE-mRNAs of these 5 miRNAs (hsa-miR-485-3p, hsa-miR-486-5p, hsa-miR-933, hsa-miR-551a, and hsa-miR-1224-5p) was presented in a heatmap (see Figure 5(b)).

### 3.5. Functional Enrichment Analysis

**Figure 6:**
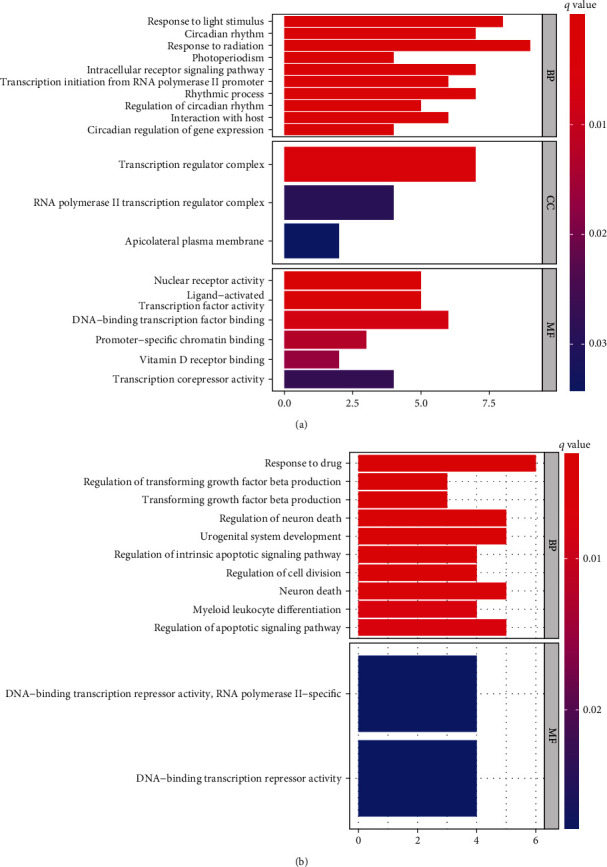
GO enrichment analysis bubble plots of DE-mRNAs targeted by five DE-miRNA factors predicting cognitive impairment in the prediction model. (a) Bubble map shows upregulated GO-enriched functional pathways. (b) Bubble diagram depicts downregulated GO-enriched functional pathways.

**Figure 7:**
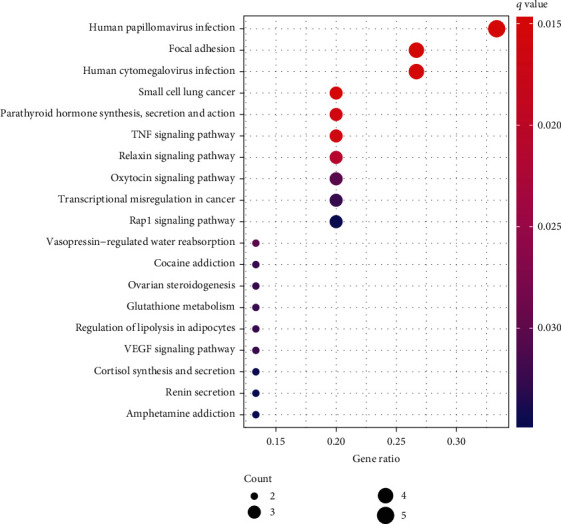
Bar graph of KEGG enrichment analysis of DE-mRNAs targeted by 5 DE-miRNA factors predicting cognitive impairment in the prediction model.

The GO and KEGG pathways were enriched to the five miRNA-targeted DE-mRNA genes in the model. The upregulated and downregulated GO pathways in cognitive impairment patients are shown in Figure 6, and the upregulated KEGG pathways are shown in Figure 7.

### 3.6. Volcano Map and Sankey

**Figure 8:**
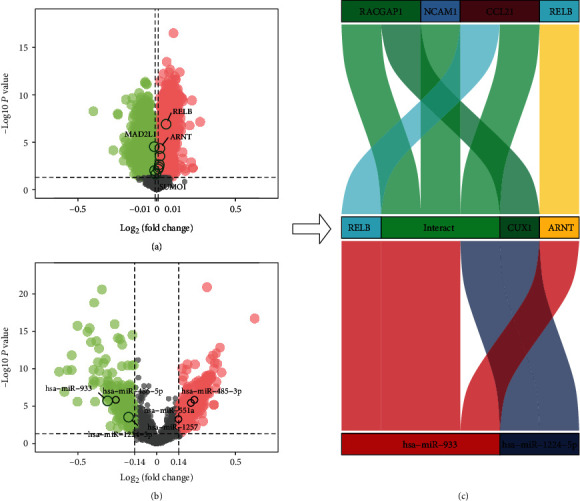
Volcano plot of DE-mRNA and DE-miRNA and Sankey plot of miRNA-TF-mRNA. (a) Volcano plots show the presence of DE-miRNAs in cognitive impairment and normal controls. The marked circles are for DE-miRNAs cooccurring in HF and cognitive impairment, present in both the cognitive impairment prediction model and the core PPI subnetwork. The miRNAs in the larger font are those miRNAs cooccurring in the cognitive impairment prediction model and the PPI subnetwork. (b) Volcano plot depicts DE-mRNA expression in cognitive impairment and normal controls. The marked circles are the DE-mRNAs targeted by miRNAs, which cooccur in HF and cognitive impairment and cooccur in the cognitive impairment prediction model and PPI subnetwork. (c) Mulberry diagram exhibits DE-miRNAs cooccurring in the model and core network and the regulatory axis of their target TF/mRNA.

The significance of miRNA differential analysis and fold change in cognitive impairment and normal controls was shown in a volcano plot (see Figure 8(a)). Figure 8 also demonstrates the differential expression of DE-miRNAs in HF and cognitive impairment both in the cognitive impairment prediction model and in the core PPI subnetwork. Some miRNAs, such as hsa-miR-933 and hsa-miR-1224-5p, were present in both the cognitive impairment prediction model and PPI subnetwork which could be found in Figure 8(b). The DE-mRNA expression in cognitive impairment and normal controls was integrated in volcano plots. Furthermore, the DE-mRNAs targeted by the DE-miRNAs shared by both the cognitive impairment prediction model and PPI subnetwork were labelled. In Table 5, the results of the differential analysis of the two miRNAs mentioned above and their targeted DE-mRNAs could be found. The miRNA-TF-mRNA axes of these 2 miRNAs and their target genes were presented in a mulberry map (see Figure 8(c)).

### 3.7. GSEA of Core Genes

**Figure 9:**
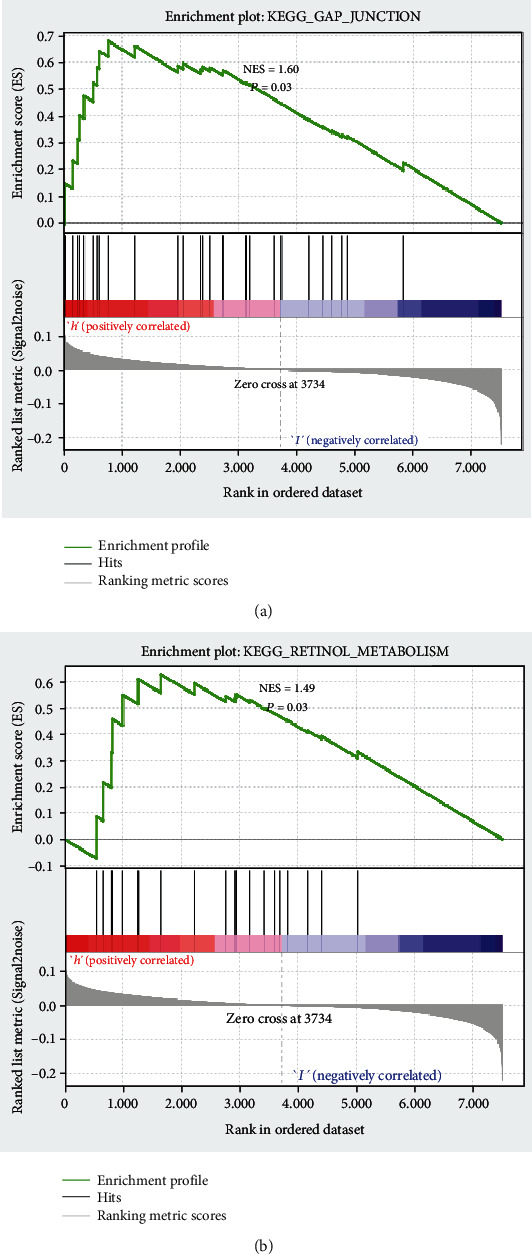
The target DE-mRNAs of miRNAs were selected according to the prediction model and subnetwork, and the TF RELB with the largest difference ploidy is selected for GSEA. The results show that the higher RELB expression group is enriched on KEGG_RETINOL_METABOLISM (*P* = 0.032) and KEGG_GAP_JUNCTION (*P* = 0.032).

The mulberry map was constructed based on the targeted DE-mRNA of miRNAs selected. Combined with Table 5, the transcription factor RELB with the largest differential fold was determined, and the hsa-miR-933/RELB/CCL21 regulatory axis was developed, according to the prediction model and subnetwork. GSEA was performed on RELB in high- and low-expression groups (see Figures 9(a) and 9(b)). The results showed that the RELB high-expression group was enriched to KEGG_RETINOL_METABOLISM (*P* = 0.032) and KEGG_GAP_JUNCTION (*P* = 0.032), suggesting that the role of the regulatory axis may be similar in the development of HF and cognitive impairment.

## 4. Discussion

The treatment of HF is highly challenging in modern medicine. The present study was the first to investigate the correlation between HF and cognitive disorders from the perspective of miRNA-mRNA via a potential vascular-neural pathway. The crosstalk between HF and cognitive impairment as well as the underlying mechanisms was comprehensively investigated. This work will generate fresh insight into the theoretical basis for clinical translation by demonstrating that the hsa-miR-933/RELB/CCL21 regulatory axis played an important role in HF and cognitive disorders.

In this study, based on the GEO dataset, serum DE-miRNAs and DE-mRNAs in brain tissue from cognitive impairment patients and normal controls were obtained. Similarly, corresponding subjects were also acquired from HF patients and their controls. DE-miRNAs and DE-mRNAs present in both HF and cognitive impairment were detected to map a PPI network jointly associated with the two diseases. At the same time, a logistic regression model for predicting cognitive impairment incidence was established and further validated by comparing the results with clinical observations; here, the prediction model was verified to have a strong predictive performance. Moreover, the key miRNAs in the logistic regression model and the core TF in the PPI subnetwork were used to build a miRNA-TF-mRNA pathway. Further GSEA on the cores was performed, and the retinol metabolism and gap junction pathways were found to play similar regulatory roles in the development of HF and cognitive impairment.

The hsa-miR-933/RELB/CCL21 regulatory axis was speculated to function critically in HF and cognitive disorders. Also, the intron microRNA hsa-miR-933 is potentially associated with the development of neurodegenerative diseases and diabetes, and its important role in regulating ATF2 target genes could explain the observed association to some extent [23]. In addition, the miR-933 expression is correlated with numerous cancers, including oral squamous cell carcinoma, breast cancer, and colon cancer [24–26]. RELB, a TF for NF-kappaB, plays an important function in endothelial cells [27], which are vital components of the circulatory system and are partially involved in the development of HF and cognitive impairment [28]. The study also indicated the active role of RELB in neurodevelopment and central nervous system functions [29]. Our enrichment analysis revealed that the group with higher RELB expression was enriched to retinol metabolism and gap junction. In the present study, RELB as a TF regulating CCL21 expression was found to be possibly regulated by hsa-miR-933. Moreover, a previous study observed that RELB is positively correlated with CCL21 expression in dendritic cells [30, 31]. In cardiac tissues, CCL21 is considered a possible biomarker for the development of HF [32]. Previous findings demonstrated a regulatory role of chemokines vital in physiological or pathological conditions of the central nervous system [33]. As a well-studied neuronal chemokine, the pathological expression of CCL21 has been detected in cerebral ischemia [34], axonal injury [35], amyotrophic lateral sclerosis [36], and spinal cord injury [37]. Thus, hsa-miR-933, RELB, and CCL21 may be correlated with HF and neurological disorders.

Previous studies have found that miRNAs in circulating blood alone also have the potential to predict dementia and HF [38–40]. This is because the information in circulating blood miRNAs is suggestive of essential organismal conditions such as ischemic, cardiomyopathy, diabetes, and valvular [41–44]. Thus, heart failure may influence the development of dementia by affecting miRNA expression in the blood microenvironment, a speculation that was first tentatively confirmed in this study. Moreover, severity of HF was found to be associated with the expression profile of circulating blood miRNAs [45]. Notably, miR-485-3p was also found to be potentially relevant to severity of HF in a previous study [46]. High expression of miR-485-3p was also found to be associated with dementia risk in the present study. In this research, the chi-squared test also showed that the severity of heart failure was significantly associated with the risk of developing cognitive impairment; that is, as the degree of heart failure increased, the risk of developing cognitive impairment increased, which is consistent with the results of previous studies [47, 48]. Thus, this study suggested that the severity of HF is also related to dementia. In addition, miR-486-5p was also found to be a biomarker for the development of heart failure and dementia in a previous study [49, 50]. In conclusion, the present study is a preliminary one, and although we identified some interesting targets, further studies are necessary in the future.

In this study, based on the GEO dataset, DE-miRNAs and DE-mRNAs present in both HF and cognitive impairment were acquired and analysed. A logistic regression model with a high performance in predicting cognitive impairment incidence was established using the DE-miRNAs. The core miRNA-TF-mRNA pathway was built by mapping the PPI network jointly associated with cognitive impairment and HF. Moreover, GSEA showed that RELB as a core TF was enriched in retinol metabolism and gap junction pathways. Investigating the potential relevancy of the hsa-miR-933/RELB/CCL21 in more clinical samples is highly necessary in the future. We will also explore the mechanisms of the hsa-miR-933/RELB/CCL21 regulatory axis in the development of HF and cognitive disorders by performing animal and cellular experiments.

## 5. Conclusion

In summary, this study was the first to examine the crosstalk between HF and cognitive impairment and the underlying mechanisms applying bioinformatics analysis. Based on PPI networks, the hsa-miR-933/RELB/CCL21 regulatory axis was considered a potential culprit in the development of both HF and cognitive disorders. The current findings provide a theoretical and experimental basis, but the mechanisms of the hsa-miR-933/RELB/CCL21 regulatory axis in the development of HF and neurological disorders should be validated by cellular and animal experiments.

## Figures and Tables

**Table 1:** Results of miRNA enrichment.

miRNA enrichment item	Percentage of genes	*P* value
Regulation of nucleobase, nucleoside, nucleotide, and nucleic acid metabolism (biological process)	25	0.044
Nucleus (cellular component)	56	0.007
Transcription factor activity (molecular function)	11	0.032
Integrin-linked kinase signaling (biological pathway)	27	0.041

**Table 2:** Uni- and multilogistic regression analyses for predicting cognitive impairment.

Variables	Unilogistic regression			Multilogistic regression		
	*β*	Odds ratio (95% CI)	*P* value	*β*	Odds ratio (95% CI)	*P* value
Age	0.183	1.201 (1.173-1.231)	*P* ≤ 0.001	0.185	1.204 (1.174-1.236)	*P* ≤ 0.001
Sex	-0.783	0.457 (0.352-0.592)	*P* ≤ 0.001	-0.510	0.6 (0.442-0.816)	0.001
hsa-miR-636	-0.678	0.508 (0.401-0.637)	*P* ≤ 0.001	0.099	1.104 (0.826-1.471)	0.499
hsa-miR-485-3p	0.387	1.472 (1.255-1.726)	*P* ≤ 0.001	0.340	1.405 (1.121-1.765)	0.003
hsa-miR-486-5p	-0.373	0.689 (0.591-0.803)	*P* ≤ 0.001	-0.263	0.769 (0.6-0.985)	0.038
hsa-miR-933	-0.301	0.74 (0.652-0.839)	*P* ≤ 0.001	-0.355	0.701 (0.584-0.84)	*P* ≤ 0.001
hsa-miR-551a	0.468	1.597 (1.312-1.958)	*P* ≤ 0.001	0.299	1.348 (1.038-1.762)	0.027
hsa-miR-296-3p	-0.405	0.667 (0.55-0.806)	*P* ≤ 0.001	-0.129	0.879 (0.666-1.159)	0.361
hsa-miR-342-3p	0.359	1.432 (1.206-1.701)	*P* ≤ 0.001	0.1925	1.212 (0.962-1.531)	0.105
hsa-miR-615-3p	0.315	1.37 (1.174-1.596)	*P* ≤ 0.001	-0.0618	0.94 (0.749-1.178)	0.530
hsa-miR-1224-5p	-0.360	0.698 (0.569-0.845)	*P* ≤ 0.001	-0.2666	0.766 (0.593-0.979)	0.037
hsa-miR-1257	0.583	1.792 (1.305-2.65)	0.001	0.30883	1.362 (0.941-2.103)	0.128
hsa-miR-1246	-0.110	0.896 (0.831-0.968)	0.005	0.05926	1.061 (0.946-1.192)	0.314

Note: *β* is the regression coefficient.

**Table 3:** Prediction factors for prevalence of cognitive impairment.

Variables	Prediction model		
	*β*	Odds ratio (95% CI)	*P* value
(Intercept)	-10.561	0 (0-0)	*P* ≤ 0.001
Age	0.184	1.202 (1.172-1.234)	*P* ≤ 0.001
Sex	-0.511	0.6 (0.443-0.812)	*P* ≤ 0.001
hsa-miR-485-3p	0.368	1.445 (1.178-1.774)	*P* ≤ 0.001
hsa-miR-486-5p	-0.189	0.828 (0.678-1.011)	0.064
hsa-miR-933	-0.392	0.676 (0.575-0.792)	*P* ≤ 0.001
hsa-miR-551a	0.356	1.427 (1.123-1.827)	0.004
hsa-miR-1224-5p	-0.290	0.748 (0.591-0.939)	0.014

Note: *β* is the regression coefficient.

**Table 4:** *C*-index of the nomogram prediction model.

Dataset group	*C*-index of the prediction model	
	*C*-index	The *C*-index (95% CI)
Training set	0.816	0.788-0.843
Validation set	0.815	0.789-0.841
Entire cohort	0.812	0.725-0.899

**Table 5:** Two DE-miRNAs and nine DE-mRNAs.

id	logFC	*t*	*P* value	Adjusted *P* value	*B*
hsa-miR-933	-0.31	-4.77	*P* ≤ 0.001	*P* ≤ 0.001	4.47
hsa-miR-1224-5p	-0.18	-3.62	*P* ≤ 0.001	0.002	-0.21
RELB	0.06	5.32	*P* ≤ 0.001	*P* ≤ 0.001	5.85
MAD2L1	-0.02	-4.2	*P* ≤ 0.001	*P* ≤ 0.001	0.61
ARNT	0.02	4.10	*P* ≤ 0.001	*P* ≤ 0.001	0.19
CCL21	0.02	3.63	*P* ≤ 0.001	0.003	-1.56
CUX1	0.02	3.06	0.002	0.015	-3.48
NCAM1	0.01	2.87	0.004	0.025	-4.04
RACGAP1	0.01	2.72	0.007	0.035	-4.46
RUNX1	-0.01	-2.59	0.010	0.045	-4.78
SUMO1	-0.01	-2.27	0.023	0.087	-5.56

## Data Availability

The miRNA expression microarray data from peripheral blood of HF patients were downloaded from GSE104150 and GSE53473. Peripheral blood miRNA expression microarray data of cognitive impairment patients was downloaded from GSE120584. RNA-seq data from myocardial tissue of HF patients were downloaded from GSE116250 to obtain mRNA expression files. mRNA expression microarray data from brain tissue of cognitive impairment patients were downloaded from GSE140831. Clinical data can be obtained by contacting the corresponding author.

## Abbreviations

DE: Differentially expressed
GO: Gene Ontology
GEO: Gene Expression Omnibus
GSEA: Gene set enrichment analysis
HC: Healthy control
HF: Heart failure.

## Supplementary Materials

Supplementary Materials Supplementary Table 1: basic characteristics of patients.
