# Supplementary material for: Crosstalk between Heart Failure and Cognitive Impairment via hsa-miR-933/RELB/CCL21 Pathway
Source: Biomed Res Int. 2021 Sep 18;2021:2291899. doi: 10.1155/2021/2291899 (PMC8478533; doi:10.1155/2021/2291899)
Supplement: Supplementary Materials — Supplementary Table 1: basic characteristics of patients. [file 2291899.f1.docx]

| Supplementary Table 1. Basic characteristics of patients | | | | |
| --- | --- | --- | --- | --- |
| Variable |  | Cognitive Impairment | | |
|  |  | NO [N=40] | YES [N=55] | P-value |
| Age (year) |  | 68.53±8.12 | 74.71±8.02 | p<0.001 |
| BMI (kg/m^2^) |  | 23.60±3.83 | 22.90±3.50 | 0.356 |
| Sex | |  |  | 0.001 |
| Female |  | 15(37.50) | 40(72.73) |  |
| Male |  | 25(62.50) | 15(27.27) |  |
| Hypertension |  |  |  | 0.319 |
| NO |  | 30(75.00) | 36(65.45) |  |
| YES |  | 10(25.00) | 19(34.55) |  |
| Diabetes |  |  |  | 0.365 |
| NO |  | 28(70.00) | 43(78.18) |  |
| YES |  | 12(30.00) | 12(21.82) |  |
| Hyperlipidemia |  |  |  | 0.487 |
| NO |  | 28(70.00) | 42(76.36) |  |
| YES |  | 12(30.00) | 13(23.64) |  |
| NYHA |  |  |  | 0.02 |
| I |  | 7(17.50) | 2(3.64) |  |
| II |  | 16(40.00) | 14(25.45) |  |
| III |  | 14(35.00) | 29(52.73) |  |
| IV |  | 3(7.50) | 10(18.18) |  |
| Albumin(g/L) |  | 42.61±2.97 | 41.36±3.49 | 0.07 |
| Hemoglobin(g/L) |  | 132.07±14.36 | 130.23±13.72 | 0.527 |
| Relative expression of miRNAs |  |  |  |  |
| hsa_miR_485_3p |  | 3.07±0.78 | 3.44±0.67 | 0.015 |
| hsa_miR_486_5p |  | 6.10±0.90 | 5.76±0.76 | 0.045 |
| hsa_miR_933 |  | 3.37±0.95 | 3.03±0.85 | 0.069 |
| hsa_miR_551a |  | 2.33±0.72 | 2.69±0.67 | 0.013 |
| hsa_miR_1224_5p |  | 4.26±0.65 | 3.96±0.79 | 0.051 |
|  |  |  |  |  |
| NYHA: Classification of nyha heart function | | | | |
